# IL-36α Promoted Wound Induced Hair Follicle Neogenesis via Hair Follicle Stem/Progenitor Cell Proliferation

**DOI:** 10.3389/fcell.2020.00627

**Published:** 2020-09-02

**Authors:** Lin Gong, Jian Xiao, Xiaokun Li, Yuanhong Li, Xinghua Gao, Xuegang Xu

**Affiliations:** ^1^Department of Dermatology, The First Hospital of China Medical University, Shenyang, China; ^2^Department of Dermatology, The First Affiliated Hospital of Dalian Medical University, Dalian, China; ^3^School of Pharmaceutical Sciences, Wenzhou Medical University, Wenzhou, China

**Keywords:** hair follicle, wound epithelialization, IL-36α, stem cell, IL-6/STAT3 pathway

## Abstract

**Background:**

Wound-induced hair follicle neogenesis (WIHN) is a phenomenon of hair neogenesis that occurs at the center of a scar when the wound area is sufficiently large. Neogenic hair follicles are separated from the pre-existing follicles at the wound edge by a hairless circular region. This WIHN study provides a unique model for developing treatments for hair loss and deciphering the mechanisms underlying organogenesis in adult mammals.

**Methods:**

The skin of a mouse was wounded by excising a 1.5 × 1.5 cm^2^ square of full-thickness dorsal skin. iTRAQ technology was used to screen proteins differentially expressed between the inner and outer scar areas in a mouse model of WIHN, on post-wounding day 15, to identify the regulators of WIHN. Owing to the overexpression of interleukin-36α (IL-36α) in the *de novo* hair follicle growth area, the regulating effect of IL-36α overexpression in WIHN was investigated. Hair follicle stem/progenitor cells were counted by flow cytometry while the expression of hair follicle stem/progenitor cell markers (*Lgr5, Lgr6, Lrig1, K15*, and *CD34*) and that of Wnt/β-catenin and IL-6/STAT3 pathway intermediaries was detected by qPCR and western blotting.

**Results:**

We found that wounding induced IL-36α expression. Incorporation of recombinant murine IL-36α (mrIL-36α) into murine skin wounds resulted in a greater number of regenerated hair follicles (*p* < 0.005) and a faster healing rate. The expression of hair follicle stem/progenitor cell markers was upregulated in the mrIL-36α-injected site (*p* < 0.05). Additionally, mrIL-36α upregulated the IL-6/STAT3 pathway intermediaries.

**Conclusion:**

IL-36α is upregulated in *de novo* hair follicle growth areas and can promote wound epithelialization and WIHN.

## Introduction

Wound-induced hair follicle neogenesis (WIHN) is a phenomenon in which the skin, sebaceous glands, and hair follicles regenerate following the occurrence of large, full-thickness wound in adult mice. WIHN has also been observed in rabbits, sheep, and spiny mice (Billingham and Russell, 1956; Brook et al., 1960; Seifert et al., 2012), while there are conflicting reports about WIHN in rats (Guerrero-Juarez et al., 2018). Studying WIHN in different species is helpful for deciphering mechanisms underlying the regeneration in mammals and may provide insights into novel approaches for hair loss treatments.

In addition to the canonical Wnt/β-catenin pathway (Ito et al., 2007), inflammation-related factors are also believed to play important roles in WIHN. Macrophages regulate WIHN through the AKT/β-catenin pathway by secreting a cytokine known as tumor necrosis factor (TNF) (Wang et al., 2017). Activated γδT cells secrete FGF9 (fibroblast growth factor 9), which promotes WIHN (Gay et al., 2013). Further, interleukin 1 (IL-1), along with IL-7 can activate the T cells to promote the proliferation of hair follicle stem cells (Lee et al., 2017). The IL-6/STAT3 (signal transducers and activators of transcription 3) axis, which is induced in response to tissue damage and double-stranded RNA production, also promotes WIHN (Nelson et al., 2015).

Interleukin-36α is a pro-inflammatory cytokine of the IL-1 family. It acts on both epithelial cells and specific immune cells, by inducing cellular activation and secretion of cytokines and chemokines, leading to the recruitment and activation of a variety of immune cells (Bassoy et al., 2018). Using iTRAQ (isobaric tags for relative and absolute quantitation) technology, we analyzed the differential protein expression in the inner and outer scar regions in a WIHN mouse model. We found that IL-36α was upregulated in the center of the scar, which is the main area for *de novo* hair follicle growth. Previous studies demonstrated that inflammation-related factors, such as IL-1, IL-6, and TNF, could promote WIHN. IL-36α has been proven to induce the secretion of these inflammation-related factors (Dietrich et al., 2016). Accordingly, we evaluated the effects of IL-36α with respect to promoting WIHN and investigated the underlying pathways responsible for regulating the hair growth.

## Materials and Methods

### Animal Model

Mice with a C57BL/6 background were purchased from Liaoning Changsheng Biotechnology Co., Ltd. (Benxi, China). Female mice (7–10 weeks old) were used in all *in vivo* experiments. All animal procedures were performed ethically in accordance with the institutional guidelines for animal experiments and was approved by the Institutional Animal Care and Use Committee at China Medical University, China.

### WIHN Surgical Procedure

Mice were anesthetized *via* isoflurane inhalation (RWD Life Science Co., Shenzhen, China) and had their dorsal hair shaved off. Skin wounds were introduced by excising a 1.5 × 1.5 cm^2^ square of full-thickness dorsal skin (used for investigating wound healing and related hair neogenesis as indicated). Subsequently, either 1 ng/μL recombinant murine IL-36α (mrIL-36α; R&D systems, Minneapolis, MN, United States) – dissolved in 50 μL phosphate-buffered-saline (PBS) – or PBS alone (Solarbio, Beijing, China) was injected into the healing wound (beneath the scab into the granulation tissue) once a day for post-wound day (PWD) 7–14. Full-thickness healed skin was harvested at PWD 15 and divided into two groups – the *de novo* hair follicle area (inner area) and no hair follicle area (outer area) – and used for proteomic analysis, real time quantitative PCR (qPCR), and western blotting for IL-36α expression detection. Full-thickness healed skin from wounded mice was harvested at PWD 7, 10, 12, 15, and 20, and normal skin from non-wounded mice was used for immunohistochemistry, qPCR, and western blotting to detect the spatiotemporal expression of IL-36α.

In addition, 50 μL of 1 ng/μL mrIL-36α – dissolved in PBS – or PBS only was injected intradermally into two separate dorsal skin regions of 7-week-old mice once a day. Full-thickness injected skin was harvested for qPCR and western blotting at day 3- and flow cytometry at day 4-post injection.

### Hair Follicle Quantification

Healed skin was collected at PWD 28 and incubated in 5% dispase (Gibco, Carlsbad, CA, United States) overnight at 25 ± 1°C. The epidermis was removed under a stereoscopic microscope (ZSA302,COIC, Chongqing, China) and the remaining dermis was fixed in 10% buffered formalin for 10 min, rinsed thrice with PBS and incubated with BCIP/NBT (BCIP/NBT Alkaline Phosphatase Color Development Kit, Jiangsu, China) for 30 min. Lastly, the dermal preparation was rinsed twice with distilled water to stop the reaction. Hair follicle quantification was performed under a dissecting microscope.

### Proteomic Analysis

In order to meet the minimum sample size and to minimize potential confounding factors in iTRAQ experiments, we pooled 15 mice for our proteomic analyses. Experiments were performed in triplicate, and only proteins with confirmed identification in all three experiments have been reported here. The proteins were extracted, resolved by sodium dodecyl-sulfate polyacrylamide gel electrophoresis (SDS-PAGE) on a 10% gel, and visualized using Coomassie Blue staining to check for protein quality. The protein samples (200 μg) were mixed with dl-dithiothreitol (DTT) and alkylated with iodoacetamide (IAA). Subsequently, samples were subjected to repeated ultrafiltration by UA (urea, Tris–HCl, pH 8) twice, tetraethyl-ammonium bromide (TEAB) thrice, and treated with trypsin overnight at a trypsin-to-protein ratio of 1:50 at 37°C.

Peptides (100 μg) from each group were labeled using an iTRAQ reagent-8 plex multiplex kit (AB SCIEX, Framingham, MA, United States). One microliter sample was tested from each group to analyze labeling and extraction efficiency. All samples were pooled and vacuum-dried. Next, the mixed lyophilized peptides were resuspended in solution A (20 mM ammonium formate, pH 10) and supernatants were collected after centrifugation at 12,000 × *g* for 20 min. Subsequently, the samples were loaded onto C18 reversed-phase columns (Michrom Bioresources, Inc., Auburn, CA, United States) and eluted with a non-linear gradient ramped from 0 to 90% mobile phase B (phase A: 20 mM ammonium formate, pH 10, phase B: 20 mM ammonium formate in ACN, pH 10) at a 0.7 μL/min flow rate for 53 min. After 5 min, the eluates were collected at 1.5 min intervals. A total of 60 fractions were collected and subsequently combined into six fractions according to peak intensities. Supernatants were collected after centrifuging the collected fractions at 14,000 × *g* for 10 min for analysis.

Peptides were analyzed using an ultra-performance liquid chromatography–mass spectrometry/mass spectrometry (LC-MS/MS) platform. The LC separation was performed on an Easy nLC 1000 (Thermo Fisher Scientific, Waltham, MA, United States) with an in-house packed capillary column (150 μm I.D. × 12 cm) with 1.9 μm C18 reverse-phase fused-silica (Michrom Bioresources). The sample was eluted with a non-linear gradient ramped from 8 to 95% mobile phase B (phase A: 0.1% formic acid in water, phase B: 0.1% FA in ACN) at a 0.6 μL/min flow rate for 78 min.

The raw mass data for the peptide data analysis were processed using the Proteome Discoverer Software with a false discovery rate (FDR) ≤ 0.01 whilst searching the Uniprot-mouse_170221.fasta database. Protein with at least two unique peptides were identified. The upregulated or downregulated proteins in both replicates with relative quantification *p* < 0.05 and 1.5-fold changes (FC) were selected to be the differentially expressed proteins (DEPs).

### Real Time Quantitative PCR (qPCR)

Total RNA from the full-thickness murine skin was extracted using a miRNeasy mini kit (Qiagen, Hilden, Germany) following the manufacturer’s instructions. cDNA was synthesized from RNA using a GoScript Reverse Transcription System (Promega, Madison, WI, United States). qPCR was performed in 96-well plates using a 7900HT Fast qPCR system (Applied Biosystems, Foster City, CA, United States); *GAPDH* expression was used as the internal control to normalize the expression of the target genes; fold change in expression was calculated using the ΔΔCt method. Primer sequences used to amplify the murine genes are listed in Supplementary Table S1.

### Total Skin Epithelial Cell Isolation and Flow Cytometry Analysis

Subcutaneous fat was mechanically removed, and the dorsal skins were incubated in dispase (2 mg/mL) for 40 min at 37°C. Epidermis was harvested by mechanical scraping with a scalpel and incubated in digestive fluid [0.05% Trypsin (Gibco), 100 μg/mL DNase (Lablead, Beijing, China)] for 40 min at 37°C. An equivalent volume of complete medium was then added to stop the digestion. Cells were strained through a 70 μm cell filter (Solarbio), and the resulting solution was centrifuged at 1000 × *g* for 8 min. The supernatant was discarded and the remaining adult mouse-derived primary skin epithelial cells (MPSECs) were washed twice with PBS (containing 1% fetal calf serum).

Single cell suspensions of MPSECs in PBS were incubated with anti-mCD49f-FITC (BioLegend, United States), anti-mCD34-PE (BD Pharmingen, Franklin Lakes, NJ, United States), and anti-mP-cadherin-APC (R&D systems) for 25 min at 4°C. After washing twice, the cells were resuspended in PBS. FACS analyses were performed using a Flow Cytometer (BD LSRFortessa, BD Biosciences, Franklin Lakes, NJ, United States).

### Western Blotting

Total protein was extracted in RIPA lysis buffer (Beyotime Biotechnology, Haimen, China) supplemented with protease inhibitors and phosphatase inhibitors (Beyotime Biotechnology). Protein concentration was determined using the BCA assay (Thermo Fisher Scientific). Protein samples of 30 μg were separated by SDS-PAGE (Thermo Fisher Scientific) and transferred to polyvinylidene difluoride (PVDF) membranes (Beyotime Biotechnology). The membranes were incubated with anti-mIL-36α (1:1000, R&D systems), anti-mpSTAT3 (1:2000, Cell Signaling Technology, Danvers, MA, United States) and anti-mSTAT3 (1:2000, Cell Signaling Technology) overnight at 4°C, then incubated with horseradish peroxidase-conjugated secondary antibodies for 2 h at 25 ± 1°C. The protein bands were visualized using BeyoECL Plus (Beyotime Biotechnology).

### Immunohistochemistry

Full-thickness skin tissue was fixed using 10% formaldehyde, embedded in paraffin, and cut into 4-μm-thick sections. The sections were deparaffinized in xylene and then rehydrated using an alcohol series. Following which, the sections were incubated with the anti-IL-36α antibody (1:500; Abcam, Cambridge, United Kingdom) overnight at 4°C. The sections were then incubated with a secondary antibody at 25 ± 1°C for 30 min, followed by incubation with a diaminobenzidine tetrachloride solution at 25 ± 1°C for 1 min and counterstained with Mayer’s hematoxylin. Images were captured using an optical microscope (Olympus, Tokyo, Japan).

### Statistical Analysis

All statistical analyses were performed using SPSS 18.0 (IBM, Armonk, NY, United States). Differential RNA expression levels were compared using a Wilcoxon matched pair test and the Student’s *t*-test was used to analyze the differences between groups. A two-sided *p* < 0.05 was considered statistically significant in all analyses.

## Results

### Proteins Differentially Expressed Between *de novo* Hair Follicles Areas and No Hair Follicle Areas Within the Scar Region

**FIGURE 1:**
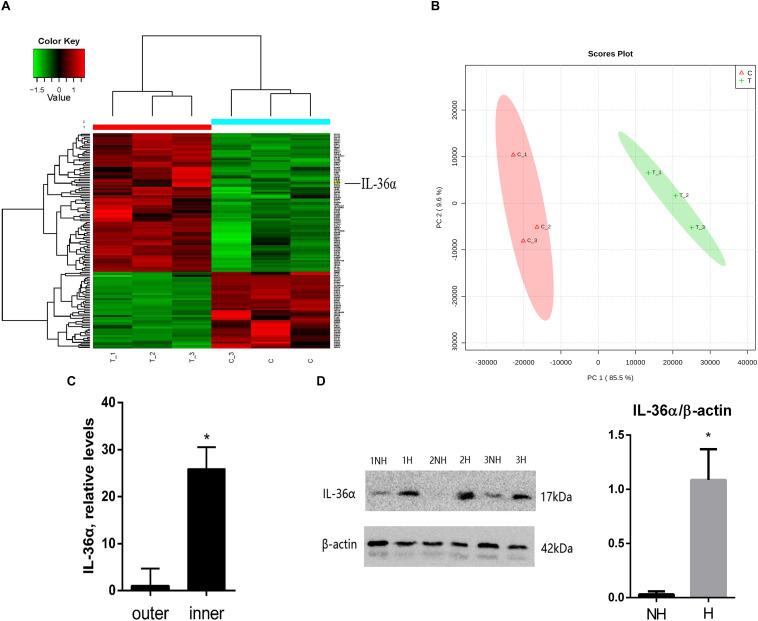
Proteins differentially expressed between *de novo* hair follicle areas and no hair follicle areas within the scar region with upregulation of IL-36α in the inner area of the wound. **(A)** Heat map representing 129 differentially expressed proteins between *de novo* hair follicle areas (inner) and no hair follicle areas (outer) within the scar region. **(B)** Principal Component Analysis (PCA) reveals distinct separation between tissue types (inner and outer scar areas). Real-time quantitative PCR (qPCR) **(C)** and western blot **(D)** analyzed the difference of IL-36α expression between inner (H = *de novo* hair follicle area) and outer (NH = no hair follicle area) scar areas at post-wound day (PWD) 15 (*n* = 3; *p < 0.05).

We replicated the WIHN model as described by Ito et al., 2007, and observed that all *de novo* hair follicles grew in the center of the scar. In the WIHN mouse model, neogenic hair follicles appeared within a fairly narrow time window of between PWD 14–21 (Ito et al., 2007; Fan et al., 2011). In order to identify the factors that promote hair follicle growth at the scar center, we analyzed DEPs between the inner and outer areas of the scar at PWD 15 using iTRAQ technology (Supplementary Figure S1). A total of 4,378 proteins were identified and 129 DEPs were detected among the inner and outer scar areas with 46 upregulated and 83 downregulated proteins in outer area vs. inner area (Supplementary Table S2). Data are available via ProteomeXchange with identifier PXD012868 (Figures 1A,B). We performed a gene ontology (GO^1^) bioinformatics analysis on the DEPs and observed that 28 proteins upregulated in the inner area were involved in biological regulation. Among these, 10 proteins were involved in signaling, eight in developmental processes, and three in inflammatory responses (Table 1).

**TABLE 1:** Proteomic analysis showing 28 upregulated proteins in the inner area (*de novo* hair follicle growth area) that are involved in biological regulation.

Genes	Fold change (inner vs. outer)	*P*-value	Biological process		
			Signaling	Developmental process	Inflammatory response
*Tnn*	2.262	0.000		✓	
*Grb10*	2.147	0.004	✓		
*Krt19*	2.035	0.007	✓	✓	
*Pawr*	1.880	0.028	✓	✓	
*Txndc9*	1.821	0.026			
*IL-36*α	1.800	0.009			✓
*Gsto1*	1.795	0.001			
*Krt6a*	1.789	0.000	✓	✓	
*Yod1*	1.783	0.003	✓		
*Rhoc*	1.740	0.043	✓	✓	
*Sptlc1*	1.708	0.037			
*Prkd3*	1.675	0.015	✓		
*Eps8l2*	1.669	0.033			
*Klk7*	1.648	0.004			
*Tab1*	1.642	0.028	✓	✓	
*Hist1h3b*	1.633	0.007			
*Aif1*	1.624	0.012	✓		✓
*Dek*	1.599	0.001			
*Hnrnpa0*	1.598	0.002			✓
*Stfa1*	1.596	0.001			
*Arg1*	1.589	0.006		✓	
*Edf1*	1.589	0.008		✓	
*Crabp1*	1.578	0.003			
*Taf5l*	1.571	0.001			
*Glrx*	1.571	0.025			
*Igf2bp2*	1.530	0.018			
*Ferritin*	1.513	0.038			
*Eif1*	1.502	0.012			

### IL-36α Is Upregulated in Inner Area of Wound

**FIGURE 2:**
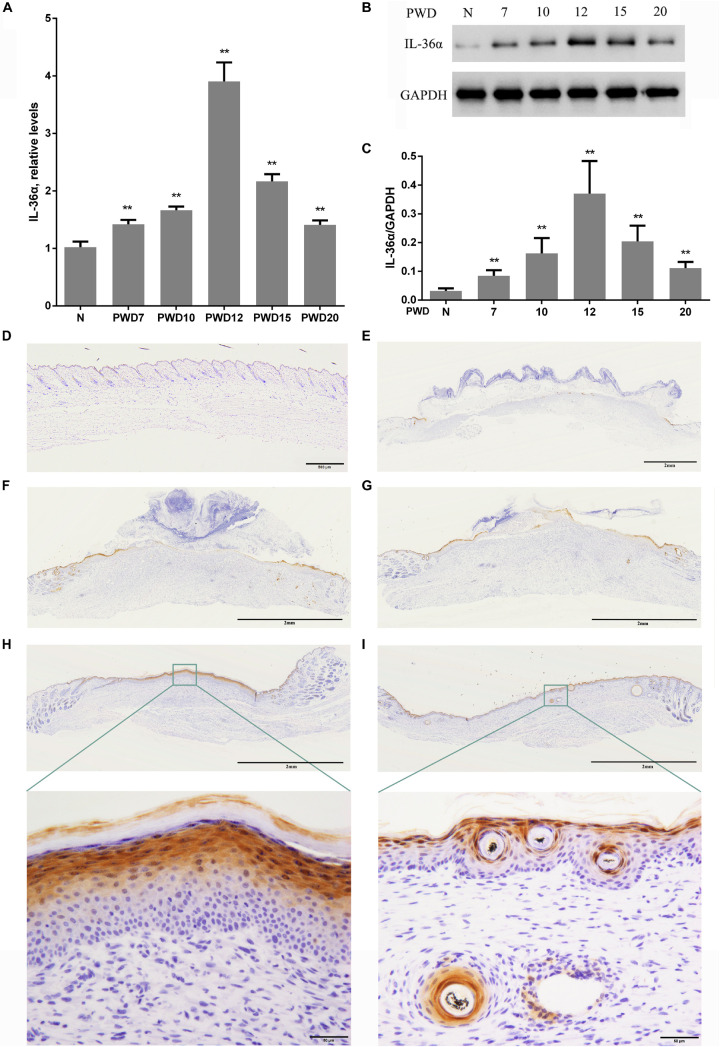
Wound-induced spatiotemporal expression of IL-36α. qPCR **(A)** and western blotting **(B,C)** for IL-36α expression at the wound area from PWD 7–20 and normal mouse skin (control). **(D–I)** Immunohistochemistry of wounded and unwounded mouse skin paraffin sections to detect IL-36α in the wound area and wound edge at PWD 0 **(D)**, 7 **(E)**, 10 **(F)**, 12 **(G)**, 15 **(H)**, and 20 **(I)** (*n* = 5, **p < 0.005, N, normal skin; PWD, post-wound day).

Considering that inflammatory factors play an important role in WIHN (Gay et al., 2013; Nelson et al., 2015; Wang et al., 2017), we focused on the activity of IL-36α (also called IL-1F6). The results of the proteomic analysis showed that IL-36α was upregulated in the inner scar area with FC = 1.799. As predicted, our data showed that IL-36α expression at the mRNA and protein levels was significantly increased in the inner scar area at PWD 15 (Figures 1C,D). Furthermore, the spatiotemporal expression pattern of IL-36α was analyzed. qPCR (Figure 2A) and western blotting (Figures 2B,C) revealed an increase in the IL-36α expression in the wound area during wound healing until PWD 12, before it gradually declined. Immunohistochemistry showed that the expression of IL-36α followed a specific spatiotemporal pattern during epithelialization. Low expression of IL-36α was observed in the normal epidermis, but higher levels of IL-36α were noted at edge of the wound at PWD 7. In addition, the expression of IL-36α was increased in the *de novo* epidermis along with wound healing. Following re-epithelialization, IL-36α was predominantly localized in the inner wounded area (compared to the outer wounded area) at PWD 15. At PWD 20, the expression of IL-36α was decreased to a normal level in the center of the wounded area where *de novo* hair follicles were present (Figures 2D–I).

### IL-36α Promotes Wound Epithelialization and Wound Induced Hair Follicle Neogenesis

**FIGURE 3:**
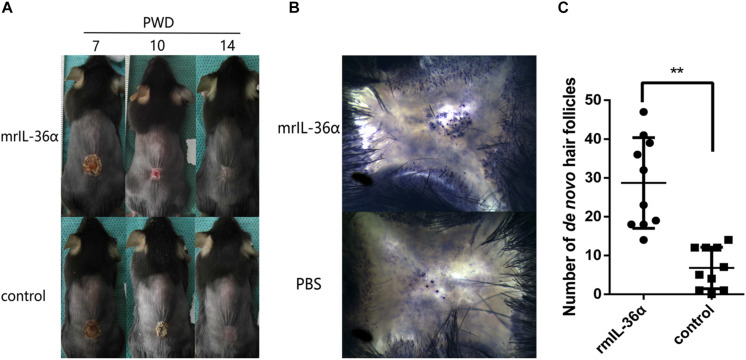
IL-36α promotes wound epithelialization and wound induced hair follicle neogenesis. A full-thickness wound of 1.5 × 1.5 cm^2^ square was made. A total of 50 μL of 1 ng/μL mrIL-36α dissolved in PBS or PBS only was injected into the healing wound (beneath the scab into the granulation tissue) from post-wound day (PWD) 7–14. **(A)** Wound model photographs of murine recombinant IL-36α (mrIL-36α)-injected group (*n* = 10) and control group (*n* = 10). **(B)** *De novo* hair follicles in skin stained for alkaline phosphatase activity at PWD 28. **(C)** *De novo* hair follicle counting in wild type mice after single dose of mrIL-36α (50 ng) compared to PBS control (**p < 0.005, *n* = 20).

To explore the function of IL-36α in WIHN, we injected 50 ng mrIL-36α (dissolved in PBS) or PBS into the wound area (beneath the scab) from PWD 7–14. In the mrIL-36α-treated group, the scab sloughed off at around PWD 10, which remained present in the control group until around PWD 12–14 (Figure 3A and Supplementary Figure S2). Moreover, the addition of mrIL-36α into murine skin wounds led to a greater number of regenerated hair follicles (average 29 hair follicles) compared to the control group (average seven hair follicles) (Figures 3B,C).

### IL-36α Causes an Increase in the Expression of Hair Follicle Stem/Progenitor Cell Markers

**FIGURE 4:**
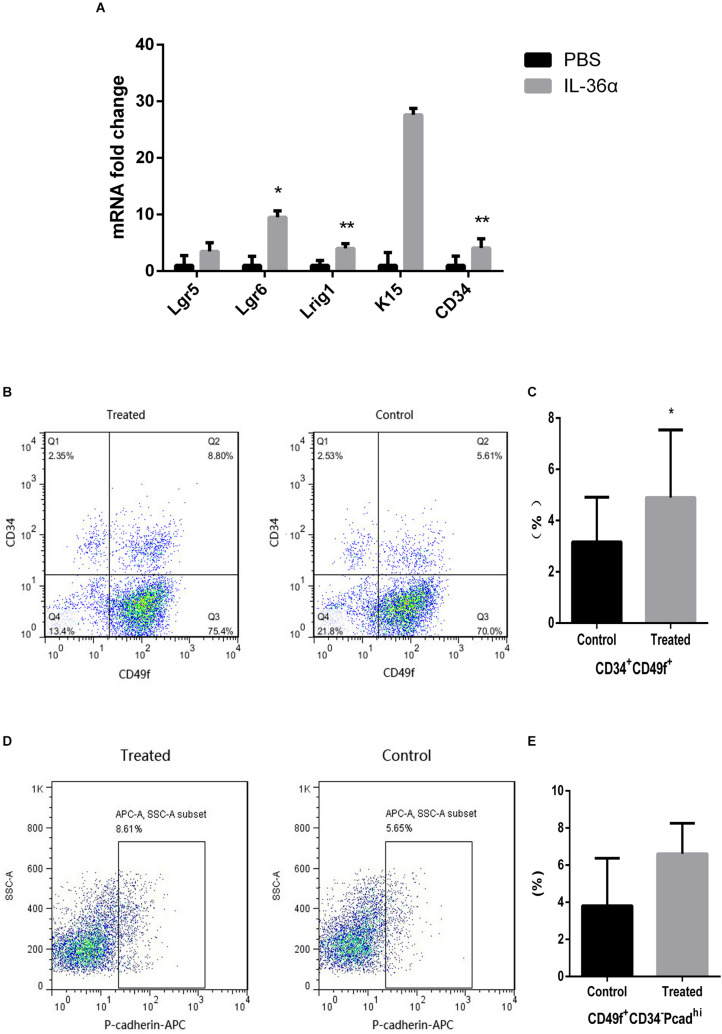
IL-36α causes an increase in the expression of hair follicle stem/progenitor cell markers in mice skin. A total of 50 μL of 1 ng/μL mrIL-36α dissolved in PBS or PBS only was injected intradermally into two separate dorsal regions of skin of 7-week-old mice for 3 days. **(A)** The relative mRNA expression levels of hair follicle stem/progenitor cell markers (*Lgr5, Lgr6, Lrig1, K15*, and *CD34*) in mrIL-36α-injected region and control region were evaluated using real time quantitative PCR (*n* = 4). Total epithelial cells were subjected to flow cytometry analysis after staining for CD34, CD49f and Pcad. Flow cytometry analysis of hair follicle stem/progenitor cells marked by CD34^+^CD49f^+^ **(B,C)** and CD34^–^ CD49f^+^Pcad^*hi*^ **(D,E)** (*p < 0.05, **p < 0.005, *n* = 4).

Quantitative PCR analyses showed increased expression of *Lgr5, Lgr6, Lrig1, K15*, and *CD34* in the mrIL-36α-injected group. Among these, the increase in *Lgr6, Lrig1*, and *CD34* were statistically significant, when compared to the controls (Figure 4A). Flow cytometry results showed an increased number of CD49f^+^CD34^+^ cells were observed in the mrIL-36α-injected region compared to the vehicle-injected region (Figures 4B,C). Although the difference in hair germ cells between the two groups was not statistically significant, a higher number of CD34^–^CD49f^+^Pcad^*hi*^ cells was observed in the IL-36α-treated group (Figures 4D,E).

### IL-36α Upregulates β-Catenin and IL-6/STAT3 Pathway Intermediaries

**FIGURE 5:**
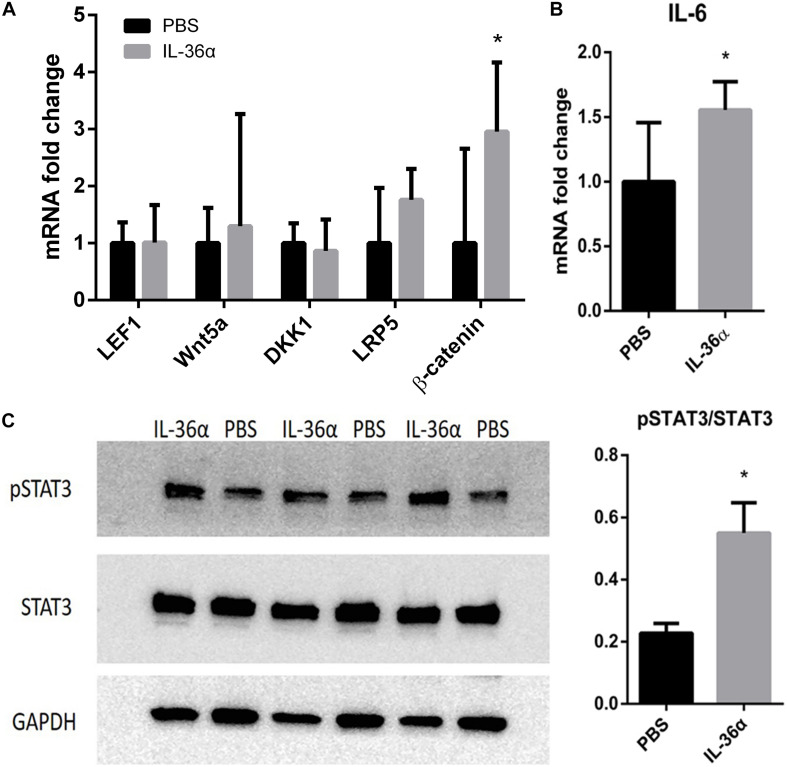
mrIL-36α causes increased expression level of β*-catenin* and *IL-6* and activates STAT3. A total of 50 μL of 1 ng/μL mrIL-36α dissolved in PBS or PBS only was injected intradermally into two separate dorsal regions of skin of 7-week-old mice for 3 days. **(A,B)** Total mRNA was isolated from mrIL-36α-injected region and control region, and mRNA levels of *LEF1, Wnt5a, DKK1, LRP5*, β*-catenin* (key genes in Wnt/β-catenin pathway) and *IL-6* were analyzed by qPCR (*n* = 4). **(C)** The phosphorylation level of STAT3 in the IL-36α-treated region and the control region analyzed by western blot (*p < 0.05, *n* = 3).

Wnt/β-catenin pathway was thought to play a vital role in hair growth and development. Although our qPCR analysis showed that IL-36α failed to regulate the expression of the key factors in Wnt pathway, β*-catenin* was highly expressed in the IL-36α-injected regions (Figure 5A). A previous work has demonstrated that the IL-6/STAT3 signaling pathway promotes WIHN (Nelson et al., 2015). Our qPCR analysis showed increased mRNA levels of *IL-6* in the mrIL-36α-injected regions compared to the controls (Figure 5B). Western blot analysis showed increased phosphorylated levels of STAT3 in IL-36α-treated regions when compared to the control regions (Figure 5C).

## Discussion

Although inflammation plays an important role in hair growth and regeneration, its exact effect on hair growth and regeneration remains unclear. Inhibiting inflammation facilitates the treatment of alopecia areata (Strazzulla et al., 2018). Many studies have shown that inflammatory immune cells, such as CD4^+^ and CD8^+^ T cells, lead to dystrophic hair follicle cycling with premature entry into the telogen phase in alopecia areata-affected mice or human models (Strazzulla et al., 2018). There is no existing evidence to show that inhibiting inflammation can treat androgenic alopecia, although more inflammatory molecules were found in skin regions affected by androgenic alopecia areas than the unaffected areas (Bao et al., 2017). However, previous WIHN studies have shown that inflammation-related factors are capable of promoting *de novo* hair follicle growth (Gay et al., 2013; Nelson et al., 2015; Wang et al., 2017). In the present study, we identified many inflammation-related factors upregulated in new hair growth scar areas, including *IL-36*α (*Il1f6*), *Aif1* (Wang et al., 2013), *Pawr*, *Yod1* (Sehrawat et al., 2013), *Stk11ip*, and *Nlrp10* (Damm et al., 2016), with the fold change in IL-36α being the highest. According to Wang et al. (2015) full re-epithelialization coincides with the onset of HF neogenesis and on PWD 13–14 (Wang et al., 2015). IL-36α upregulation was initiated at the edge of the wound and gradually progressed to the inner wounded area. Following re-epithelialization, upregulation of IL-36α in the inner wounded area was higher than that in the outer wounded area. Our study demonstrated that wound induced IL-36α expression, which in turn gradually increased the expression of IL-36α with reepithelization. Further experiments demonstrated that IL-36α enhanced the IL-6/STAT3 pathway and the expression of hair follicle stem/progenitor cell markers, and significantly impacted WIHN and wound healing. Therefore, we predicted that inflammation was crucial for organ neogenesis, and different inflammation-related factors may have different effects on hair growth, development and regeneration.

Interleukin-36α is highly expressed in psoriatic lesions (Boutet et al., 2016), and it plays a significant role in driving the imiquimod-associated psoriatic skin pathology (Milora et al., 2015). Although population studies have revealed that patients with psoriasis have an odds ratio (OR) of 2.5 for developing alopecia areata (Wu et al., 2012), there is no evidence showing that psoriasis directly causes alopecia as the hair of patients with scalp psoriasis may be normal. Imiquimod can induce psoriasis-like skin lesions and is therefore commonly used to establish mouse psoriasis models. Amberg et al. (2016) found that imiquimod treatment during late telogen can induce hair follicle stem cell activation and premature hair cycle entry. STAT3 is also highly phosphorylated in the psoriasis lesions (Calautti et al., 2018). Activation of STAT3 can promote wound healing, keratinocytes migration, hair follicle growth and participate in tumor occurrence (Sano et al., 2008). STAT3 phosphorylation has been shown to promote the proliferation of hair follicle stem/progenitor cells (Rao et al., 2015) and WIHN (Nelson et al., 2015). Our results show that IL-36α can upregulate the IL-6/STAT3 pathway, further induce hair follicle stem/progenitor cell activation and WIHN. Together with previous studies, these data suggest that increased IL-36α in patients with psoriasis does not directly induce alopecia. Although IL-36α upregulated β-catenin it failed to regulate the expression of the key genes in the Wnt pathway. A previous study had demonstrated that AKT/β-catenin signaling but not Wnt/β-catenin pathway was crucial for TNF-induced hair follicle neogenesis. Although our study does not address the mechanism of IL-36α-driven β-catenin regulation, it will be interesting to obtain further insight into how IL-36α induces β-catenin and IL-6/STAT3 signaling and how the negative feedback is regulated.

Stem cell activation is essential for wound epithelialization and hair follicle growth. Ito et al. (2007) pointed out that stem cells in the epidermis and upper portion of the follicle (infundibulum) are mainly involved in *de novo* hair follicles formation after wounding (Ito et al., 2007). However, a recent study by Wang et al. (2017) reported the crucial role of Lgr5^+^ hair follicle stem cells in WIHN (Wang et al., 2017). Hair follicle stem cells in the bulge region can be identified by many proteins, such as K15, Lgr5, and CD34 (Cotsarelis et al., 1990; Ito et al., 2005; Plikus et al., 2012). Lgr6 is one of the earliest placode markers and its stem cells located in the hair follicle isthmus are thought to give rise to hair follicle, sebaceous gland, and inter-follicular epidermal lineage, and have been verified to promote WIHN (Snippert et al., 2010; Lough et al., 2016). Lrig1^+^ stem cells tend to contribute predominantly to infundibulum and sebaceous gland lineages and only occasionally to the inter-follicular epidermis (Jensen et al., 2009). CD34^+^CD49f^+^ stem cells are the most studied as hair follicle stem/progenitor cells in mice (Ouji et al., 2010). In addition, hair germ cells can be identified as CD34^–^CD49f^+^Pcad^*hi*^ (Greco et al., 2009), which is the principal source of the rapidly proliferating cells that contribute to the initial stages of hair follicle regeneration. From our data, we learnt that IL-36α promotes hair follicle stem/progenitor cell proliferation.

Hair follicles are the smallest, complete anatomical organs that have the ability to regenerate. Following a full-thickness wound, the hair follicle in the wound area was thought to be lost entirely. However, hair follicle neogenesis occurred after reepithelization at around PWD 14–15. Therefore, we chose the time point of PWD 15 to assay DEPs between the hair-growth area and non-hair-growth area, in order to identify the regulators of hair follicle neogenesis. We collected the skin to count the number of *de novo* hair follicles at PWD 28, as mature anagen *de novo* hair follicles were present and entered the first telogen phase at around PWD 35 (Wang et al., 2015). In our study, the WIHN model presents a phenomenon of *de novo* hair follicle growth without pigmentation at the center of scar, irrespective of whether it is treated with mrIL-36α or PBS. Similar to previous studies, wound can induce an embryonic-like hair follicle regeneration (Gong et al., 2018). As we know, the survival rate of hair transplantation in scars are lower than that of the normal skin. Future studies can focus on the microenvironment and growth situation of the *de novo* hair follicles induced by wound, which could provide aid to improving the survival rate of hair transplantation.

In conclusion, inflammation appears to play an important role in hair follicle neogenesis. In particular, IL-36α is upregulated in scar areas with *de novo* hair follicular growth, and can promote WIHN, which may induce hair follicle stem/progenitor cell proliferation and secretion of inflammation-related factors. Further research on WIHN will contribute to a better understanding of hair neogenesis and new insights into the treatment of hair-related disorders.

## Data Availability Statement

The datasets generated for this study can be found in the ProteomeXchange with identifier PXD012868.

## Ethics Statement

The animal study was reviewed and approved by the Institutional Animal Care and Use Committee at China Medical University.

## Author Contributions

XG and XX designed this study. LG and XX performed the experiments and analyzed the data. LG drafted the manuscript. JX and XL revised the manuscript for important intellectual content. All authors have read and approved the final manuscript.

## Conflict of Interest

The authors declare that the research was conducted in the absence of any commercial or financial relationships that could be construed as a potential conflict of interest.

## Footnotes

1 http://www.geneontology.org

## References

[B1] Amberg N. Holcmann M. Stulnig G. Sibilia M. (2016). Effects of imiquimod on hair follicle stem cells and hair cycle progression. *J. Invest. Dermatol.* 136 2140–2149. 10.1016/j.jid.2016.06.613 27377695

[B2] Bao L. Yu A. Luo Y. Tian T. Dong Y. Zong H. (2017). Genomewide differential expression profiling of long non-coding RNAs in androgenetic alopecia in a Chinese male population. *J. Eur. Acad. Dermatol. Venereol.* 31 1360–1371. 10.1111/jdv.14278 28419572

[B3] Bassoy E. Y. Towne J. E. Gabay C. (2018). Regulation and function of interleukin-36 cytokines. *Immunol. Rev.* 281 169–178. 10.1111/imr.12610 29247994

[B4] Billingham R. E. Russell P. S. (1956). Incomplete wound contracture and the phenomenon of hair neogenesis in rabbits’ skin. *Nature* 177 791–792. 10.1038/177791b0 13321965

[B5] Boutet M. A. Bart G. Penhoat M. Amiaud J. Brulin B. Charrier C. (2016). Distinct expression of interleukin (IL)-36alpha, beta and gamma, their antagonist IL-36Ra and IL-38 in psoriasis, rheumatoid arthritis and Crohn’s disease. *Clin. Exp. Immunol.* 184 159–173. 10.1111/cei.12761 26701127 PMC4837235

[B6] Brook A. H. Short B. F. Lyne A. G. (1960). Formation of new wool follicles in the adult sheep. *Nature* 185:51. 10.1038/185051a0 13804752

[B7] Calautti E. Avalle L. Poli V. (2018). Psoriasis: a STAT3-centric view. *Int. J. Mol. Sci.* 19:171. 10.3390/ijms19010171 29316631 PMC5796120

[B8] Cotsarelis G. Sun T. T. Lavker R. M. (1990). Label-retaining cells reside in the bulge area of pilosebaceous unit: implications for follicular stem cells, hair cycle, and skin carcinogenesis. *Cell* 61 1329–1337. 2364430 10.1016/0092-8674(90)90696-c

[B9] Damm A. Giebeler N. Zamek J. Zigrino P. Kufer T. A. (2016). Epidermal NLRP10 contributes to contact hypersensitivity responses in mice. *Eur. J. Immunol.* 46 1959–1969. 10.1002/eji.201646401 27221772

[B10] Dietrich D. Martin P. Flacher V. Sun Y. Jarrossay D. Brembilla N. (2016). Interleukin-36 potently stimulates human M2 macrophages, Langerhans cells and keratinocytes to produce pro-inflammatory cytokines. *Cytokine* 84 88–98. 10.1016/j.cyto.2016.05.012 27259168

[B11] Fan C. Luedtke M. A. Prouty S. M. Burrows M. Kollias N. Cotsarelis G. (2011). Characterization and quantification of wound-induced hair follicle neogenesis using in vivo confocal scanning laser microscopy. *Skin Res. Technol.* 17 387–397. 10.1111/j.1600-0846.2011.00508.x 21492240 PMC3164919

[B12] Gay D. Kwon O. Zhang Z. Spata M. Plikus M. V. Holler P. D. (2013). Fgf9 from dermal gammadelta T cells induces hair follicle neogenesis after wounding. *Nat. Med.* 19 916–923. 10.1038/nm.3181 23727932 PMC4054871

[B13] Gong L. Xu X. G. Li Y. H. (2018). Embryonic-like regenerative phenomenon: wound-induced hair follicle neogenesis. *Regen. Med.* 13 729–739. 10.2217/rme-2018-0028 30255731

[B14] Greco V. Chen T. Rendl M. Schober M. Pasolli H. A. Stokes N. (2009). A two-step mechanism for stem cell activation during hair regeneration. *Cell Stem Cell* 4 155–169. 10.1016/j.stem.2008.12.009 19200804 PMC2668200

[B15] Guerrero-Juarez C. F. Astrowski A. A. Murad R. Dang C. T. Shatrova V. O. Astrowskaja A. (2018). Wound regeneration deficit in rats correlates with low morphogenetic potential and distinct transcriptome profile of epidermis. *J. Invest. Dermatol.* 138:1409. 10.1016/j.jid.2017.12.030 29317265 PMC6059613

[B16] Ito M. Liu Y. Yang Z. Nguyen J. Liang F. Morris R. J. (2005). Stem cells in the hair follicle bulge contribute to wound repair but not to homeostasis of the epidermis. *Nat. Med.* 11 1351–1354. 10.1038/nm1328 16288281

[B17] Ito M. Yang Z. Andl T. Cui C. Kim N. Millar S. E. (2007). Wnt-dependent de novo hair follicle regeneration in adult mouse skin after wounding. *Nature* 447 316–320. 10.1038/nature05766 17507982

[B18] Jensen K. B. Collins C. A. Nascimento E. Tan D. W. Frye M. Itami S. (2009). Lrig1 expression defines a distinct multipotent stem cell population in mammalian epidermis. *Cell Stem Cell* 4 427–439. 10.1016/j.stem.2009.04.014 19427292 PMC2698066

[B19] Lee P. Gund R. Dutta A. Pincha N. Rana I. Ghosh S. (2017). Stimulation of hair follicle stem cell proliferation through an IL-1 dependent activation of gammadeltaT-cells. *eLife* 6:e28875. 10.7554/eLife.28875 29199946 PMC5714500

[B20] Lough D. M. Wetter N. Madsen C. Reichensperger J. Cosenza N. Cox L. (2016). Transplantation of an LGR6+ epithelial stem cell-enriched scaffold for repair of full-thickness soft-tissue defects: the in vitro development of polarized hair-bearing skin. *Plast. Reconstr. Surg.* 137 495–507. 10.1097/01.prs.0000475761.09451.00 26818284

[B21] Milora K. A. Fu H. Dubaz O. Jensen L. E. (2015). Unprocessed Interleukin-36alpha Regulates Psoriasis-Like Skin Inflammation in Cooperation With Interleukin-1. *J. Invest. Dermatol.* 135 2992–3000. 10.1038/jid.2015.289 26203636 PMC4648684

[B22] Nelson A. M. Reddy S. K. Ratliff T. S. Hossain M. Z. Katseff A. S. Zhu A. S. (2015). dsRNA released by tissue damage activates TLR3 to drive skin regeneration. *Cell Stem Cell* 17 139–151. 10.1016/j.stem.2015.07.008 26253200 PMC4529957

[B23] Ouji Y. Yoshikawa M. Nishiofuku M. Ouji-Sageshima N. Kubo A. Ishizaka S. (2010). Effects of Wnt-10b on proliferation and differentiation of adult murine skin-derived CD34 and CD49f double-positive cells. *J. Biosci. Bioeng.* 110 217–222. 10.1016/j.jbiosc.2010.01.020 20547359

[B24] Plikus M. V. Gay D. L. Treffeisen E. Wang A. Supapannachart R. J. Cotsarelis G. (2012). Epithelial stem cells and implications for wound repair. *Semin. Cell Dev. Biol.* 23 946–953. 10.1016/j.semcdb.2012.10.001 23085626 PMC3518754

[B25] Rao D. Macias E. Carbajal S. Kiguchi K. DiGiovanni J. (2015). Constitutive Stat3 activation alters behavior of hair follicle stem and progenitor cell populations. *Mol. Carcinog.* 54 121–133. 10.1002/mc.22080 24038534 PMC4139480

[B26] Sano S. Chan K. S. DiGiovanni J. (2008). Impact of Stat3 activation upon skin biology: a dichotomy of its role between homeostasis and diseases. *J. Dermatol. Sci.* 50 1–14. 10.1016/j.jdermsci.2007.05.016 17601706

[B27] Sehrawat S. Koenig P. A. Kirak O. Schlieker C. Fankhauser M. Ploegh H. L. (2013). A catalytically inactive mutant of the deubiquitylase YOD-1 enhances antigen cross-presentation. *Blood* 121 1145–1156. 10.1182/blood-2012-08-447409 23243279 PMC3575758

[B28] Seifert A. W. Kiama S. G. Seifert M. G. Goheen J. R. Palmer T. M. Maden M. (2012). Skin shedding and tissue regeneration in African spiny mice (Acomys). *Nature* 489 561–565. 10.1038/nature11499 23018966 PMC3480082

[B29] Snippert H. J. Haegebarth A. Kasper M. Jaks V. van Es J. H. Barker N. (2010). Lgr6 marks stem cells in the hair follicle that generate all cell lineages of the skin. *Science* 327 1385–1389. 10.1126/science.1184733 20223988

[B30] Strazzulla L. C. Wang E. H. C. Avila L. Lo Sicco K. Brinster N. Christiano A. M. (2018). Alopecia areata: an appraisal of new treatment approaches and overview of current therapies. *J. Am. Acad. Dermatol.* 78 15–24. 10.1016/j.jaad.2017.04.1142 29241773

[B31] Wang J. Zhao Y. Wang W. Du Z. Yan D. Li C. (2013). Daintain/AIF-1 plays roles in coronary heart disease via affecting the blood composition and promoting macrophage uptake and foam cell formation. *Cell Physiol. Biochem.* 32 121–126. 10.1159/000350130 23867161

[B32] Wang X. Chen H. Tian R. Zhang Y. Drutskaya M. S. Wang C. (2017). Macrophages induce AKT/beta-catenin-dependent Lgr5+ stem cell activation and hair follicle regeneration through TNF. *Nat. Commun.* 8:14091. 10.1038/ncomms14091 28345588 PMC5378973

[B33] Wang X. Hsi T. C. Guerrero-Juarez C. F. Pham K. Cho K. McCusker C. D. (2015). Principles and mechanisms of regeneration in the mouse model for wound-induced hair follicle neogenesis. *Regeneration* 2 169–181. 10.1002/reg2.38 26504521 PMC4617665

[B34] Wu J. J. Nguyen T. U. Poon K. Y. Herrinton L. J. (2012). The association of psoriasis with autoimmune diseases. *J. Am. Acad. Dermatol.* 67 924–930. 10.1016/j.jaad.2012.04.039 22664308

