# Supplementary material for: IL-36α Promoted Wound Induced Hair Follicle Neogenesis via Hair Follicle Stem/Progenitor Cell Proliferation
Source: Front Cell Dev Biol. 2020 Sep 2;8:627. doi: 10.3389/fcell.2020.00627 (PMC7493638; doi:10.3389/fcell.2020.00627)
Supplement: Supplementary file 1 [file Data_Sheet_1.pdf]

Supplementary Material

1 Supplementary Figures

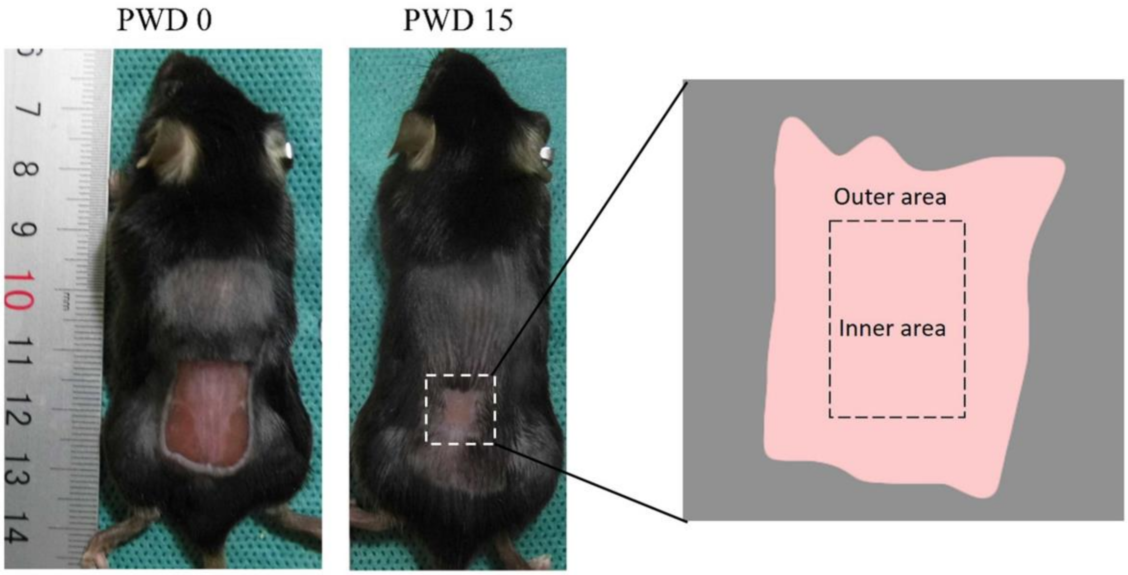

Supplementary Figure 1. Wound model photos at post-wound day (PWD) 0 and PWD 15. Inner and outer regions were separated along the black dashed line.

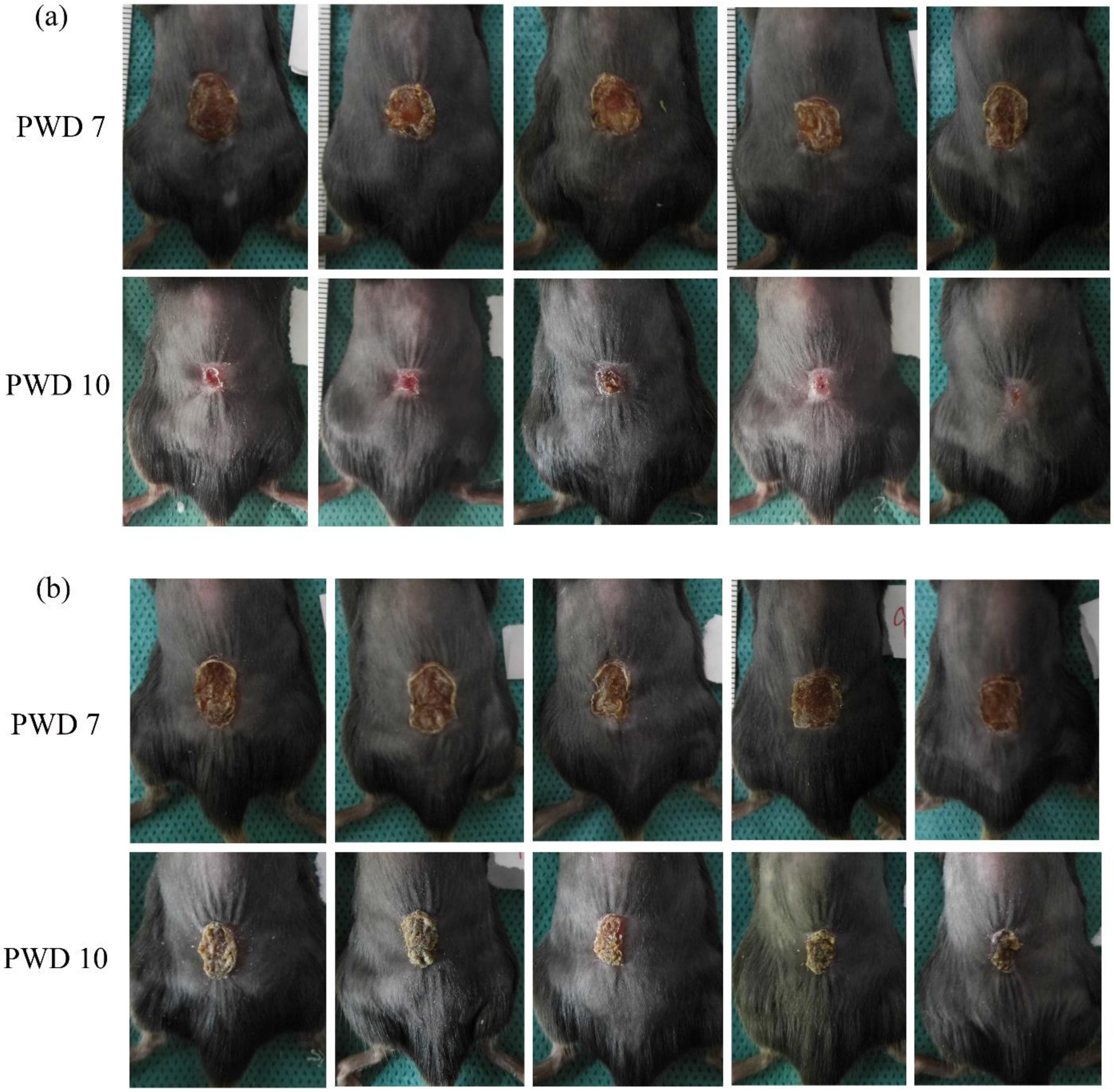

Supplementary Figure 2. mrIL-36α or PBS was injected into healing wounds (beneath the scab) from PWD 7 to PWD 14. Wound model photos of mrIL-36α-injected group (a) and control group (b).

2 Supplementary Tables

Supplementary Table 1. Primer sequences used for qPCR are provided.

Gene	Primer sequence
Lgr5	Forward: CCTACTCGAAGACTTACCCAGT
	Reverse: GCATTGGGGTGAATGATAGCA
Lgr6	Forward: TCAGGGAACCACCTCTCACA
	Reverse: CATAGTGCCTCTGCTGGGAT
Lrig1	Forward: TTGAGGACTTGACGAATCTGC
	Reverse: CTTGTTGTGCTGCAAAAAGAGAG
K15	Forward: AGATCGGGACTACAGCCATTAC
	Reverse: AGGGTCAGCTCATTCTCATACT
CD34	Forward: AAGGCTGGGTGAAGACCCTTA
	Reverse: TGAATGGCCGTTTCTGGAAGT
IL-36α	Forward: GCAGCATCACCTTCGCTTAGA
	Reverse: CAGATATTGGCATGGGAGCAAG
IL-6	Forward: CTGCAAGAGACTTCCATCCAG
	Reverse: AGTGGTATAGACAGGTCTGTTGG

Supplementary Table 2. Differentially expressed proteins (DEPs) between the inner and outer areas of the scar at PWD 15.

Accession	Gene Name	Description	FC	*P* value
Q61726	Krt83	Keratin type II (Fragment) OS=Mus musculus GN=Krt83 PE=2 SV=1	4.875278	0.012515
Q6IFZ6	Krt77	Keratin, type II cytoskeletal 1b OS=Mus musculus GN=Krt77 PE=1 SV=1	4.695016	0.001437
Q8VCW2	Krt25	Keratin, type I cytoskeletal 25 OS=Mus musculus GN=Krt25 PE=1 SV=1	4.690756	0.014254
Q8VED5	Krt79	Keratin, type II cytoskeletal 79 OS=Mus musculus GN=Krt79 PE=1 SV=2	4.386128	0.000993
B7ZN13	Aldh3a1	Aldehyde dehydrogenase OS=Mus musculus GN=Aldh3a1 PE=2 SV=1	3.465496	0.000316
Q05A13	Sdr16c6	Short-chain dehydrogenase/reductase family 16C member 6 OS=Mus musculus GN=Sdr16c6 PE=2 SV=1	3.306859	0.000439
Q9R0H5	Krt71	Keratin, type II cytoskeletal 71 OS=Mus musculus GN=Krt71 PE=1 SV=1	2.62385	0.012513
Q7TNT2	Far2	Fatty acyl-CoA reductase 2 OS=Mus musculus GN=Far2 PE=2 SV=1	2.522687	2.69E-05
Q9QWL7	Krt17	Keratin, type I cytoskeletal 17 OS=Mus musculus GN=Krt17 PE=1 SV=3	2.503422	0.000464
E9QK82	Mpz	Myelin protein P0 OS=Mus musculus GN=Mpz PE=1 SV=1	2.425743	0.017215
Q3UFX6	Mgll	Putative uncharacterized protein OS=Mus musculus GN=Mgll PE=2 SV=1	2.333964	0.000464
Q9D1H9	Mfap4	Microfibril-associated glycoprotein 4 OS=Mus musculus GN=Mfap4 PE=1 SV=1	2.19034	1.55E-05
A0A140LHD6	Serpinh1	Serpin H1 (Fragment) OS=Mus musculus GN=Serpinh1 PE=1 SV=1	2.16251	0.010913
B2RU72	Krt27	Keratin 27 OS=Mus musculus GN=Krt27 PE=2 SV=1	2.161607	0.023779

Q8R0F9	Sec14l4	SEC14-like protein 4 OS=Mus musculus GN=Sec14l4 PE=1 SV=1	2.000684	0.002855
Q9EQR4	Clca3a2	Chloride channel calcium activated 2 OS=Mus musculus GN=Clca3a2 PE=1 SV=1	1.980923	0.000331
Q5F2E4	Alox12e	Arachidonate lipoxygenase, epidermal OS=Mus musculus GN=Alox12e PE=2 SV=1	1.933724	0.001578
Q3TNA0	Cyp4b1	MCG1260 OS=Mus musculus GN=Cyp4b1 PE=1 SV=1	1.924366	2.74E-05
Q3UIR3	Dtx3l	E3 ubiquitin-protein ligase DTX3L OS=Mus musculus GN=Dtx3l PE=1 SV=1	1.862903	0.00301
Q8BRM1	Faah	Putative uncharacterized protein OS=Mus musculus GN=Faah PE=2 SV=1	1.850082	0.009373
P07310	Ckm	Creatine kinase M-type OS=Mus musculus GN=Ckm PE=1 SV=1	1.839134	0.015723
P54320	Eln	Elastin OS=Mus musculus GN=Eln PE=1 SV=2	1.814499	0.00042
Q678L1	Krt77	Keratin 1b (Fragment) OS=Mus musculus GN=Krt77 PE=2 SV=1	1.791234	0.024315
Q924Y0	Bbox1	Gamma-butyrobetaine dioxygenase OS=Mus musculus GN=Bbox1 PE=1 SV=1	1.770669	1.23E-05
Q543S0	Prelp	Proline arginine-rich end leucine-rich repeat OS=Mus musculus GN=Prelp PE=1 SV=1	1.759674	9.79E-05
A0A0B6VJJ9	Klk10	Kallikrein related-peptidase 10 OS=Mus musculus GN=Klk10 PE=2 SV=1	1.756238	5.77E-05
O35233	Aff1	MAF4 (Fragment) OS=Mus musculus GN=Aff1 PE=2 SV=1	1.749513	0.015991
Q3UMM7	Adh7	Putative uncharacterized protein OS=Mus musculus GN=Adh7 PE=2 SV=1	1.73014	6.35E-06
P31428	Dpep1	Dipeptidase 1 OS=Mus musculus GN=Dpep1 PE=1 SV=2	1.726985	0.014108
E9PVG8	9530053A07 Rik	Protein 9530053A07Rik OS=Mus musculus GN=9530053A07Rik PE=1 SV=1	1.686411	0.030853

E9Q9C6	Fcgbp	Protein Fcgbp OS=Mus musculus GN=Fcgbp PE=1 SV=1	1.677492	0.001655
Q9EQC4	Elovl4	Elongation of very long chain fatty acids protein 4 OS=Mus musculus GN=Elovl4 PE=1 SV=2	1.651468	0.002227
Q9WVH9	Fbln5	Fibulin-5 OS=Mus musculus GN=Fbln5 PE=1 SV=1	1.634456	5.02E-05
E9QPZ3	Flg2	Filaggrin-2 OS=Mus musculus GN=Flg2 PE=1 SV=1	1.629405	0.020832
E9Q4S3	Nin	Ninein OS=Mus musculus GN=Nin PE=1 SV=1	1.62158	0.011784
Q9QUJ7	Acsl4	Long-chain-fatty-acid--CoA ligase 4 OS=Mus musculus GN=Acsl4 PE=1 SV=2	1.610979	0.036452
P06330	#N/A	Ig heavy chain V region AC38 205.12 OS=Mus musculus PE=1 SV=1	1.58366	0.010799
Q69ZX3	Myh11	MKIAA0866 protein (Fragment) OS=Mus musculus GN=Myh11 PE=2 SV=1	1.583033	7.73E-05
O35452	Tnxb	Protein Tnxb OS=Mus musculus GN=Tnxb PE=1 SV=1	1.554796	0.000624
Q9D9V3	Echdc1	Ethylmalonyl-CoA decarboxylase OS=Mus musculus GN=Echdc1 PE=1 SV=2	1.547578	0.00466
Q3T9U8	Entpd2	Putative uncharacterized protein OS=Mus musculus GN=Entpd2 PE=2 SV=1	1.539953	0.000236
Q9D486	Cmip	C-Maf-inducing protein OS=Mus musculus GN=Cmip PE=1 SV=3	1.512938	0.023893
P16015	Ca3	Carbonic anhydrase 3 OS=Mus musculus GN=Ca3 PE=1 SV=3	1.511782	0.025686
J3QJX6	7530416G11 Rik	Protein 7530416G11Rik OS=Mus musculus GN=7530416G11Rik PE=4 SV=1	1.508008	0.007932
P11087	Col1a1	Collagen alpha-1(I) chain OS=Mus musculus GN=Col1a1 PE=1 SV=4	1.507353	0.022273
Q3UKR1	Dcn	Decorin OS=Mus musculus GN=Dcn PE=1 SV=1	1.500471	5.61E-05

P48024	Eif1	Eukaryotic translation initiation factor 1 OS=Mus musculus GN=Eif1 PE=1 SV=2	0.665893	0.01209
P50518	Atp6v1e1	V-type proton ATPase subunit E 1 OS=Mus musculus GN=Atp6v1e1 PE=1 SV=2	0.665278	0.019672
Q5EBQ6	Rpl9	MCG10266, isoform CRA_a OS=Mus musculus GN=Rpl9 PE=1 SV=1	0.661645	0.003201
Q3TJJ6	#N/A	Ferritin OS=Mus musculus PE=2 SV=1	0.660886	0.03792
E9PZF0	Gm20390	Nucleoside diphosphate kinase OS=Mus musculus GN=Gm20390 PE=3 SV=1	0.654797	0.017303
Q3UJX2	Ctnnbl1	Putative uncharacterized protein OS=Mus musculus GN=Ctnnbl1 PE=2 SV=1	0.654774	0.00156
Q3UJR3	Htra2	Putative uncharacterized protein OS=Mus musculus GN=Htra2 PE=2 SV=1	0.654442	0.005789
Q08189	Tgm3	Protein-glutamine gamma-glutamyltransferase E OS=Mus musculus GN=Tgm3 PE=1 SV=2	0.654181	0.000431
Q5SF07	Igf2bp2	Insulin-like growth factor 2 mRNA-binding protein 2 OS=Mus musculus GN=Igf2bp2 PE=1 SV=1	0.653552	0.017908
Q3UK85	Dctn5	Putative uncharacterized protein OS=Mus musculus GN=Dctn5 PE=2 SV=1	0.651784	0.011431
Q497E9	Rps8	40S ribosomal protein S8 OS=Mus musculus GN=Rps8 PE=1 SV=1	0.651342	0.008769
A0A1B0GSR7	Krt10	Keratin, type I cytoskeletal 10 OS=Mus musculus GN=Krt10 PE=4 SV=1	0.647743	0.00024
Q3V3Z6	Nlrp10	Putative uncharacterized protein OS=Mus musculus GN=Nlrp10 PE=2 SV=1	0.645477	0.025691
Q8CGR6	Klk13	Epidermal growth factor-binding protein type B OS=Mus musculus GN=Klk13 PE=1 SV=1	0.644475	0.003055
Q63870	Col7a1	Collagen alpha-1(VII) chain OS=Mus musculus GN=Col7a1 PE=1 SV=3	0.643207	0.00053

Q3TKJ1	Bcat1	Branched-chain-amino-acid aminotransferase OS=Mus musculus GN=Bcat1 PE=2 SV=1	0.639099	0.01116
Q3U6L3	Glrx	Glutaredoxin, isoform CRA_a OS=Mus musculus GN=Glrx PE=1 SV=1	0.636549	0.024876
Q91WQ5	Taf5l	TAF5-like RNA polymerase II p300/CBP-associated factor-associated factor 65 kDa subunit 5L OS=Mus musculus GN=Taf5l PE=2 SV=1	0.636364	0.000608
B2RVP5	H2afv	Histone H2A OS=Mus musculus GN=H2afv PE=2 SV=1	0.634942	0.015851
Q3TIT6	Dnajb1	Putative uncharacterized protein OS=Mus musculus GN=Dnajb1 PE=2 SV=1	0.634851	0.024297
P62965	Crabp1	Cellular retinoic acid-binding protein 1 OS=Mus musculus GN=Crabp1 PE=1 SV=2	0.63387	0.0026
F6XI62	Rpl7	60S ribosomal protein L7 (Fragment) OS=Mus musculus GN=Rpl7 PE=1 SV=1	0.632446	0.013962
Q9JMG1	Edf1	Endothelial differentiation-related factor 1 OS=Mus musculus GN=Edf1 PE=1 SV=1	0.629384	0.008312
Q61176	Arg1	Arginase-1 OS=Mus musculus GN=Arg1 PE=1 SV=1	0.629312	0.006303
Q8BT90	Rps17	Putative uncharacterized protein (Fragment) OS=Mus musculus GN=Rps17 PE=2 SV=1	0.627202	0.008872
P35175	Stfa1	Stefin-1 OS=Mus musculus GN=Stfa1 PE=3 SV=1	0.62676	0.000987
Q9CX86	Hnrnpa0	Heterogeneous nuclear ribonucleoprotein A0 OS=Mus musculus GN=Hnrnpa0 PE=1 SV=1	0.625791	0.002237
Q7TNV0	Dek	Protein DEK OS=Mus musculus GN=Dek PE=1 SV=1	0.625279	0.001043
Q497J0	BC100530	MCG130175, isoform CRA_b OS=Mus musculus GN=BC100530 PE=1 SV=1	0.620736	0.001019

O70200	Aif1	Allograft inflammatory factor 1 OS=Mus musculus GN=Aif1 PE=1 SV=1	0.615864	0.01212
A0A1D5RM85	Rpl18a	60S ribosomal protein L18a (Fragment) OS=Mus musculus GN=Rpl18a PE=1 SV=1	0.612479	0.002626
P84228	Hist1h3b	Histone H3.2 OS=Mus musculus GN=Hist1h3b PE=1 SV=2	0.612196	0.007319
Q80VD1	Fam98b	Protein FAM98B OS=Mus musculus GN=Fam98b PE=1 SV=1	0.610088	0.001581
Q58EA6	Rps25	MCG10725, isoform CRA_a OS=Mus musculus GN=Rps25 PE=1 SV=1	0.609779	0.013734
Q8CF89	Tab1	TGF-beta-activated kinase 1 and MAP3K7-binding protein 1 OS=Mus musculus GN=Tab1 PE=1 SV=2	0.608974	0.028342
P63163	Snrpn	Small nuclear ribonucleoprotein-associated protein N OS=Mus musculus GN=Snrpn PE=1 SV=1	0.608527	0.004916
Q9CQH1	Teddm3	Protein Teddm3 OS=Mus musculus GN=Teddm3 PE=2 SV=1	0.607219	0.007858
Q91VE3	Klk7	Kallikrein-7 OS=Mus musculus GN=Klk7 PE=2 SV=1	0.60663	0.004321
Q99K30	Eps8l2	Epidermal growth factor receptor kinase substrate 8-like protein 2 OS=Mus musculus GN=Eps8l2 PE=1 SV=1	0.599106	0.03257
Q5FWX6	Prkd3	Serine/threonine-protein kinase OS=Mus musculus GN=Prkd3 PE=1 SV=1	0.59707	0.014584
O35704	Sptlc1	Serine palmitoyltransferase 1 OS=Mus musculus GN=Sptlc1 PE=1 SV=2	0.585366	0.036854
E9QAZ2	Gm10020	Ribosomal protein L15 OS=Mus musculus GN=Gm10020 PE=3 SV=1	0.585335	0.000696
Q7TNC4	Luc7l2	Putative RNA-binding protein Luc7-like 2 OS=Mus musculus GN=Luc7l2 PE=1 SV=1	0.583698	6.99E-06
Q62159	Rhoc	Rho-related GTP-binding protein RhoC OS=Mus musculus GN=Rhoc PE=1 SV=2	0.574675	0.042721

P62918	Rpl8	60S ribosomal protein L8 OS=Mus musculus GN=Rpl8 PE=1 SV=2	0.571247	0.009997
F8WJ23	Hrnr	Hornerin OS=Mus musculus GN=Hrnr PE=1 SV=1	0.570151	0.01297
O88986	Gcat	2-amino-3-ketobutyrate coenzyme A ligase, mitochondrial OS=Mus musculus GN=Gcat PE=1 SV=2	0.560795	0.039653
Q8CB27	Yod1	Ubiquitin thioesterase OTU1 OS=Mus musculus GN=Yod1 PE=1 SV=1	0.5607	0.003363
P50446	Krt6a	Keratin, type II cytoskeletal 6A OS=Mus musculus GN=Krt6a PE=1 SV=3	0.559059	0.000238
O09131	Gsto1	Glutathione S-transferase omega-1 OS=Mus musculus GN=Gsto1 PE=1 SV=2	0.557021	0.000667
Q149U6	Il1f6	Interleukin 1 family, member 6 OS=Mus musculus GN=Il1f6 PE=2 SV=1	0.55594	0.00869
P24472	Gsta4	Glutathione S-transferase A4 OS=Mus musculus GN=Gsta4 PE=1 SV=3	0.554728	0.02225
Q9CQ79	Txndc9	Thioredoxin domain-containing protein 9 OS=Mus musculus GN=Txndc9 PE=1 SV=1	0.549148	0.025765
A2RSX5	Serpina9	Serine (Or cysteine) peptidase inhibitor, clade A (Alpha-1 antiproteinase, antitrypsin), member 9 OS=Mus musculus GN=Serpina9 PE=2 SV=1	0.548054	0.001529
P62858	Rps28	40S ribosomal protein S28 OS=Mus musculus GN=Rps28 PE=1 SV=1	0.53777	0.001374
P97347	Rptn	Repetin OS=Mus musculus GN=Rptn PE=2 SV=2	0.536662	0.003052
Q14A95	Rbm7	MCG9666, isoform CRA_d OS=Mus musculus GN=Rbm7 PE=1 SV=1	0.536101	0.002954
Q925B0	Pawr	PRKC apoptosis WT1 regulator protein OS=Mus musculus GN=Pawr PE=1 SV=2	0.531991	0.028448

Q9CQK2	Rps24	40S ribosomal protein S24 OS=Mus musculus GN=Rps24 PE=2 SV=1	0.529042	0.002714
Q3TRJ4	Krt26	Keratin, type I cytoskeletal 26 OS=Mus musculus GN=Krt26 PE=2 SV=1	0.521244	0.001841
P47963	Rpl13	60S ribosomal protein L13 OS=Mus musculus GN=Rpl13 PE=1 SV=3	0.521222	0.002965
Q3UW40	Rpl24	Putative uncharacterized protein OS=Mus musculus GN=Rpl24 PE=2 SV=1	0.519264	0.006741
B2RT71	Csta1	Cystatin A OS=Mus musculus GN=Csta1 PE=2 SV=1	0.517814	0.003277
Q6ZWX1	Rpl35a	MCG1036414 OS=Mus musculus GN=Rpl35a PE=1 SV=1	0.510879	0.001016
Q3UP23	Tmem26	Transmembrane protein 26 OS=Mus musculus GN=Tmem26 PE=2 SV=2	0.508606	0.021886
P19001	Krt19	Keratin, type I cytoskeletal 19 OS=Mus musculus GN=Krt19 PE=1 SV=1	0.491304	0.006896
Q5BLJ9	Rpl27	60S ribosomal protein L27 OS=Mus musculus GN=Rpl27 PE=1 SV=1	0.490454	0.002805
Q8C2Q7	Hnrnph1	Heterogeneous nuclear ribonucleoprotein H OS=Mus musculus GN=Hnrnph1 PE=1 SV=1	0.489125	0.005234
F6YVP7	Gm10260	Protein Gm10260 OS=Mus musculus GN=Gm10260 PE=3 SV=2	0.47389	0.002019
Q62150	Rnps1	RNA/DNA-binding protein OS=Mus musculus GN=Rnps1 PE=2 SV=1	0.470707	0.021117
Q60760	Grb10	Growth factor receptor-bound protein 10 OS=Mus musculus GN=Grb10 PE=1 SV=2	0.465816	0.004425
Q80Z71	Tnn	Tenascin-N OS=Mus musculus GN=Tnn PE=1 SV=2	0.442065	9.20E-05
Q3TAA7	Stk11ip	Serine/threonine-protein kinase 11-interacting protein OS=Mus musculus GN=Stk11ip PE=1 SV=1	0.429104	0.039768

P14115	Rpl27a	60S ribosomal protein L27a OS=Mus musculus GN=Rpl27a PE=1 SV=5	0.422836	0.000153
A0A0A6YX26	Rpl31	60S ribosomal protein L31 OS=Mus musculus GN=Rpl31 PE=1 SV=1	0.422645	0.003188
G3UWD7	Gm10269	MCG123152 OS=Mus musculus GN=Gm10269 PE=1 SV=1	0.405022	0.001514
Q9CWK0	Rpl14	Putative uncharacterized protein OS=Mus musculus GN=Rpl14 PE=2 SV=1	0.351093	0.00052
A0A140T8K6	Rpl36-ps3	60S ribosomal protein L36 OS=Mus musculus GN=Rpl36-ps3 PE=3 SV=1	0.308702	0.00456
Q0VDR7	Krt6b	Krt6b protein OS=Mus musculus GN=Krt6b PE=2 SV=1	0.300013	6.05E-05
Q6NXX2	Rnase4	Rnase4 protein OS=Mus musculus GN=Rnase4 PE=2 SV=1	0.299113	0.000681
C6EQJ5	#N/A	ASL1/3110003A17Rik fusion protein OS=Mus musculus PE=2 SV=1	0.296389	0.000416
P61255	Rpl26	60S ribosomal protein L26 OS=Mus musculus GN=Rpl26 PE=1 SV=1	0.215309	5.84E-05
A0A0G2JEK2	Crip1	Cysteine-rich protein 1 OS=Mus musculus GN=Crip1 PE=1 SV=1	0.203902	0.003853

FC：Fold change
